# *In-utero* transfer of decidualized endometrial stromal cells increases the frequency of regulatory T cells and normalizes the abortion rate in the CBA/J × DBA/2 abortion model

**DOI:** 10.3389/fimmu.2024.1440388

**Published:** 2024-09-23

**Authors:** Kayhan Zarnani, Kimia Zarnani, Nasim Maslehat-Lay, Bahman Zeynali, Sedigheh Vafaei, Mohammad-Reza Shokri, Negar Vanaki, Haleh Soltanghoraee, Ebrahim Mirzadegan, Haleh Edalatkhah, Mohammad-Mehdi Naderi, Ali Sarvari, Farnoosh Attari, Mahmood Jeddi-Tehrani, Amir-Hassan Zarnani

**Affiliations:** ^1^ School of Biology, College of Sciences, University of Tehran, Tehran, Iran; ^2^ Reproductive Immunology Research Center, Avicenna Research Institute, Academic Center for Education, Culture and Research (ACECR), Tehran, Iran; ^3^ Department of Immunology, School of Public Health, Tehran University of Medical Sciences, Tehran, Iran; ^4^ Developmental Biology Lab., School of Biology, College of Sciences, University of Tehran, Tehran, Iran; ^5^ Department of Immunology, H. Lee Moffitt Cancer Center, Tampa, FL, United States; ^6^ Reproductive Biotechnology Research Center, Avicenna Research Institute, Academic Center for Education, Culture and Research (ACECR), Tehran, Iran; ^7^ Nanobiotechnology Research Center, Avicenna Research Institute, Academic Center for Education, Culture and Research (ACECR), Tehran, Iran; ^8^ Department of Animal Biology, School of Biology, College of Sciences, University of Tehran, Tehran, Iran; ^9^ Monoclonal Antibody Research Center, Avicenna Research Institute, Academic Center for Education, Culture and Research (ACECR), Tehran, Iran

**Keywords:** abortion, CBA/J x DBA/2, decidualization, regulatory T cells, proteome, immune system

## Abstract

**Introduction:**

Failure to adequate decidualization leads to adverse pregnancy outcomes including pregnancy loss. Although there are plenty of reports underscoring immune dysfunction as the main cause of abortion in CBA/J females mated with DBA/2 males (CBA/J × DBA/2), little is known about the potential role of impaired endometrial decidualization.

**Methods:**

Endometrial stromal cells (ESCs) from CBA/J mice were *in-vitro* decidualized, and the proteome profile of the secretome was investigated by membrane-based array. CBA/J mice were perfused *In-utero* with either decidualized ESCs (C×D/D), undecidualized ESCs (C×D/ND), or PBS (C×D/P) 12 days before mating with DBA/2 males. Control mice were not manipulated and were mated with male DBA/2 (C×D) or Balb/c (C×B) mice. On day 13.5 of pregnancy, reproductive parameters were measured. *In-vivo* tracking of EdU-labeled ESCs was performed using fluorescence microscopy. The frequency of regulatory T cells (Tregs) in paraaortic/renal and inguinal lymph nodes was measured by flow cytometry. The proliferation of pregnant CBA/J splenocytes in response to stimulation with DBA/2 splenocytes was assessed by 5,6-carboxyfluorescein diacetate succinimidyl ester (CFSE) flow cytometry.

**Results:**

In C×D/D mice, the resorption rate was reduced to match that seen in the C×B group. Intrauterine perfused ESCs appeared in uterine stroma after 2 days, which remained there for at least 12 days. There was no difference in the number of implantation sites and embryo weight across all groups. The frequency of Tregs in the inguinal lymph nodes was similar across all groups, but it increased in the paraaortic/renal lymph nodes of C×D/D mice to the level found in C×B mice. No significant changes were observed in the proliferation of splenocytes from pregnant C×D/D compared to those of the C×D group in response to stimulation with DBA/2 splenocytes. Decidualization of ESCs was associated with a profound alteration in ESC secretome exemplified by alteration in proteins involved in extracellular matrix (ECM) remodeling, response to inflammation, senescence, and immune cell trafficking.

**Discussion:**

Our results showed that the deficiency of Tregs is not the primary driver of abortion in the CBA/J × DBA/2 model and provided evidence that impaired endometrial decidualization probably triggers endometrial immune dysfunction and abortion in this model.

## Introduction

Pregnancy and reproduction are complex processes and involve different stages that act in a harmonious manner. Most Eutheria use more or less the same stages for reproduction. During this process, different systems such as hormones and the immune system take action in concert with each other to control blastocyst implantation and development. The first step of a successful pregnancy is the implantation of a competent embryo in a receptive endometrium. Impairments in this critical step can cause miscarriage (1). Implantation is associated with considerable modifications in the endometrium and endometrial immune cells. Endometrial remodeling, which is known as decidualization, is associated with extensive structural and functional modification. This process is controlled by ovarian steroid hormones during the menstrual cycle, leading to a transient window of implantation, in which endometrial luminal epithelium adheres to embryonic trophectoderm (2). Decidualization results from a complex interplay of transcription factors, chemokines, growth factors, and their associated binding proteins, morphogens, cytokines, cell cycle regulators, and signaling pathways (3).

Compared with the original endometrial stromal cells, fully differentiated decidual cells have anti-inflammatory capacity and resist stress signals mediated by endometrial remodeling following embryo implantation (4, 5). Decidualized endometrial stromal cells surround the implanting embryo (6, 7) and form a cellular matrix around the fetus to protect it against maternal immune responses (8).

Human decidualization initiates at the beginning of a secretory phase of the menstrual cycle in response to steroid hormones, while decidualization in mouse endometrium commences after embryo implantation (9). The window of implantation also coincides with the accumulation of innate immune cells in the endometrium, primarily uterine natural killer (uNK) (4, 5) highlighting a link between decidualization and endometrial immune cell adaptation, a process which is needed for the establishment of maternal tolerance for a successful pregnancy.

There are plenty of reports from preclinical animal models suggesting that an impaired decidual response is associated with pregnancy loss (10, 11). However, the precise mechanisms remain to be defined. Loss of a conspicuous epigenetic stem cell signature has been demonstrated in endometrial stromal cells from endometrial biopsies of women with recurrent pregnancy loss (12) leading to a failure of endometrium plasticity to accommodate pregnancy. Decidualization also triggers uNK to eliminate senescent decidual cells (5, 13), which could otherwise induce pregnancy loss through the induction of senescence-associated secretory phenotype (SASP) in early pregnancy.

Immune cells residing in the endometrium comprise ~40% of all cells in first-trimester decidua (14–16) and undergo extensive modulation before the establishment of pregnancy and after implantation to ensure maternal tolerance to the fetal antigens (15). The eminent role of immune tolerance during pregnancy has been the basis of decades of research into immune-related basis for recurrent pregnancy loss (15). To address the classical immune theory of maternal tolerance to non-self fetus, the CBA/J × DBA/2 abortion model arose decades ago to delineate adverse pregnancy outcomes (resorption) in the face of deficiency of immune tolerance to fetal antigens (17). The value of mean resorptions (abortions)/total number of implantations (R/T) for CBA/J × DBA/2 and CBA/J × Balb/c matings is approximately 33.32% and 5.11%, respectively (18). In CBA/J × DBA/2 mating, there is a loss of cellular contact between decidual cells and cells of the ectoplacental cone beginning approximately day 7 of gestation leading to embryonic loss by days 10–12 (17).

A number of mechanisms of immune dysregulation have been described in the CBA/J × DBA/2 model to mainly strengthen the classical theory for which this abortion model first drove (17). In this context, various immunotherapeutic approaches for the prevention of abortion in this model have also been tested, some with successful results. The most intriguing experiments suggesting immune dysfunction fingerprint in the CBA/J × DBA/2 abortion model are those focused on the suboptimal generation of regulatory T cells (Tregs). These cells are produced during pregnancy under the action of ovarian steroid hormones (19). Administration of induced Tregs or CD4^+^CD25^+^ spleen and thymus cells isolated from Balb/c-mated CBA/J female normal pregnant mice to a DBA/2-mated CBA/J female in early pregnancy decreased the resorption rate (20–22), while depletion of Tregs with anti-CD25 antibody administration (23) increased the resorption rate (21) and abolished the protective effect of Treg administration.

Although all these studies turn around the original putative role of maternal immune tolerance in the establishment of a successful pregnancy, it is still unclear how and to what extent the endometrium and specifically decidualization contribute to uterine immune regulation during pregnancy.

Early (24) and later studies (25) suggest that abnormalities existing in the CBA/J decidua and its interaction with the developing embryo are the prime movers of the pathology related to these pregnancies, and this idea is supported by the fact that “reverse” mating between DBA/2 females and CBA/J males are considered normal (26). Indeed, this mating combination suffers from a fundamental disorganization of DNA methylation (25). Collectively, these studies assume that the impaired decidual reaction could be viewed as one potential cause of abortion in this mouse model. We recently provided compelling evidence showing that human endometrial stromal cells modulate the functional features of NK (27), Treg (28), and TH17 (29, 30) cells in a pregnancy-friendly manner. Indeed, decidualized endometrial stromal cells exert potent immunomodulatory capacity (30). Based on this information, we hypothesized that *in-utero* perfusion of decidualized ESCs could rescue pregnancy loss and normalize immune dysfunction in this mouse model. Our results showed that *in-utero* perfusion of *in-vitro* decidualized, but not undecidualized, ESCs could normalize the resorption rate in DBA2-mated CBAJ female mice and upregulate Tregs in uterus-draining lymph nodes. Our results suggest that impaired decidual reaction in female CBA/J mice following mating with DBA/2 males is the main driver of immune dysregulation and abortion in this model.

## Materials and methods

### Animals

Mice ranging from 8 to 12 weeks of age (female CBA/J, male DBA/2, and male Balb/c) were obtained from Pasteur Institute, Tehran, Iran. All experimental procedures done in this study were approved by the Ethics Committee of Animal Experiments of Avicenna Research Institute (approval and grant no. IR.ACECR.AVICENNA.REC.1397.020).

### Isolation of endometrial stromal cells

Isolation of mouse endometrial stromal cells was performed according to the protocol published elsewhere with some minor modifications (31). To induce estrus in non-pregnant CBA/J female mice, a subcutaneous injection of 100 µL of 17β-estradiol (E2) (Sigma, USA) solution (100 ng/100 µL) was administered for three consecutive days. The stage of the estrous cycle was determined in female CBA/J mice by conducting wet smear preparation of vaginal secretion and Papanicolaou staining (32). In some instances, uterine horn sections were prepared and stained with H&E to confirm the estrous phase. Following euthanasia, the uterine horns were carefully dissected, removing adipose and connective tissue, and washed multiple times with Ca^2+^ Mg^2+^-free Hanks’ Balanced Salt Solution (HBSS). The uterine horns were then opened longitudinally and dissected into small pieces. These pieces underwent digestion for 1 h at 37°C with 3 mL of digestion buffer (0.075 mg/mL of pancreatin and 2.5 mg/mL of trypsin). The supernatants were discarded, and the remaining tissues were washed with HBSS before undergoing a second digestion for 30 min in 3 mL of 0.5 mg/mL collagenase. The resulting cell suspension was passed through a 70-µm nylon mesh, and the collected cells were washed with HBSS plus 10% fetal bovine serum (FBS) and then resuspended in a complete DMEM medium. After 24 h, non-adherent cells were removed, and stromal cells were allowed to propagate until reaching 75% confluency. A total of 15 mice were used for the isolation of an adequate number of endometrial stromal cells needed to perform all experiments yielding an average of approximately 8 × 10^6^ ESC/mouse.

### Immunofluorescent staining

Cultured cells were detached through trypsinization and applied to adhesive slides. After air drying for 30 min, the cells were fixed with ice-cold acetone for 2 min and washed in PBS three times. Slides underwent blocking with 5% sheep serum for 30 min at 37°C, followed by incubation with mouse anti-vimentin (Santa Cruz, USA) at a concentration of 5 µg/mL at room temperature for 1 h. Following three washes in PBS-1.5% BSA, sheep anti-mouse-FITC (Sina Biotech, Iran) was added and incubated for 45 min. Nuclei were stained with DAPI (2 µg/mL) for 2 min. After three washes in PBS, the signals were detected using an epifluorescence microscope. Intracellular cytokeratin was stained with the recommended dilution of anti-cytokeratin-FITC antibody (BD Biosciences, USA). As a positive control for cytokeratin staining, human amniotic epithelial cells (hAECs) were isolated from a human amniotic membrane following a described protocol (33) and stained as mentioned above.

### *In-vitro* decidualization

Induction of *in-vitro* decidualization was performed according to the protocol published elsewhere (31). For *in-vitro* decidualization (IVD), decidualization was induced in phenol red-free DMEM/F12 supplemented with 5% charcoal-stripped (CS)-FBS. After an overnight rest incubation period in a CO_2_ incubator, the medium was changed to phenol red-free DMEM/F12 supplemented with 2% CS-FBS and 0.5 mM of 8-Br-cAMP and 1 μM of medroxyprogesterone acetate (MPA) (both from Sigma), and the culture continued for an additional 6 days. On day 3, the medium was semi-changed. Control culture wells received the same culture medium, excluding 8-Br-cAMP and MPA. At the end of the culture period, cell culture supernatants and cells were collected for proteome and RNA expression analyses, respectively.

### Quantitative real-time PCR

Quantitative real-time PCR (qRT-PCR) was conducted on total RNA extracted from cell homogenates using an RNA extraction kit according to the manufacturer’s instructions (ROJE Technologies, Iran). RNA quantification and integrity were assessed using a NanoDrop and agarose gel electrophoresis, respectively. cDNA was synthesized using a kit (ROJE Technologies). For each specimen, three PCR reactions were set up using primers for progesterone receptor (Pgr) (F: CTCCGGGACCGAACAGAGT, R: ACAACAACCCTTTGGTAGCAG), prolactin (Prl) (F: AGCCAGAAATCACTGCCACTCT, R: CAGGAGTGATCCATGCACCCATA), and β2-microglobulin (β2m) (F: ACTGACCGGCCTGTATGCTA, R: AATGTGAGGCGGGTGGTAC) as housekeeping genes. Real-time qRT-PCR kit from Takara (Japan) was used for gene amplification. The thermal profile of the Rotor-Gene thermal cycler included an initial denaturation at 95°C for 30 s, followed by 40 cycles of denaturation at 95°C (5 s), annealing at 60°C (30 s), and extension at 72°C (20 s) with a final extension at 72°C for 5 min. Primers for Pgr and Prl were designated to amplify 122-bp and 125-bp fragments, respectively. The third set of primers amplified a 124-bp segment of the β2m gene as internal control.

### Secretome protein profiling

The protein profile of endometrial stromal cells was analyzed using a mouse XL cytokine array kit, following the manufacturer’s instructions (R&D Systems, USA). Each membrane contained captured antibodies against 111 cytokines, chemokines, growth factors, and other proteins. The pool of three supernatants from cultured *in-vitro* decidualized and undecidualized CBA/J endometrial cells was applied to the membranes in two independent preparations for each condition, and spots were developed using a biotin-streptavidin-enhanced chemiluminescence (ECL) system. Signal intensities were quantified using AlphaEase software. Integrated densities of each spot were adjusted for background signal and normalized to positive control spots on each membrane, and the resulting figures were plotted in GraphPad Prism version 9.2.

### Bioinformatics analyses

All analyses were performed with R software (version 4.3.3). Before each analysis, quantified proteins were normalized and auto-scaled. For pathway enrichment, the Clusterprofiler package version 4.10.1 and ReactomePA package version 1.46.0 were used, and for obtaining GSVA scores, the GSVA package version 1.50.1 was used. The Annotationhub package version 3.10.0 was used to annotate mouse protein IDs.

### Adoptive transfer of endometrial stromal cells

For the adoptive transfer of endometrial stromal cells, five experimental groups, each consisting of five mice, were employed. In the C×D/D group, female CBA/J mice underwent surgical *in-utero* perfusion of decidualized ESCs, while mice in the C×D/ND and C×D/P groups received undecidualized ESCs or PBS, respectively, before mating with DBA/2 males. Two groups of female CBA/J mice were not manipulated and were mated with either male DBA/2 (C×D) (abortion-prone group) or Balb/c mice (C×B) (non-abortion group). After confirming the estrous stage, mice were intraperitoneally injected with 10% ketamine (50 mg/kg) and xylazine 2% (5 mg/kg). Mice were placed on their abdomen, and after shaving back hair and disinfection, a surgical incision was made approximately 1.5 cm long and 1 cm away from the spine and parallel to it in the middle of the abdomen. Intrauterine perfusion of endometrial stromal cells was performed according to the protocol we published elsewhere (34). Accordingly, 5 × 10^5^ primary culture ESCs from non-pregnant CBA/J mice (passages 2–4) suspended in 10 μL PBS was loaded in a glass micropipette (Straight, Grinded- with spike; i.d. 100 µm) attached to an Eppendorf Cell Tram^®^ Vario micromanipulation pump and injected into the top of the right uterine horn at the utero-tubal junction (UTJ), with the aid of a ×10 stereomicroscope (Nikon, SMZ 800N). For the evaluation of leakage and tracing of the medium flow in the uterus, two mice were perfused as above with 10 µL of trypan blue solution (**Supplementary Figure S1**). Mice were then treated with tramadol (10–12 mg/kg) and gentamicin at a dose of 10 mg/kg subcutaneously for 3 days after surgery. After a 12-day recovery period, female mice were placed in the cages of male mice, and the day of vaginal plug detection was considered day 0.5 of pregnancy. On day 13.5 of pregnancy, mice in each group were sacrificed, and pregnancy parameters, including the number of implantation sites, number of live embryos, abortion percentage, and fetal weight, were assessed. Indeed, the morphology of the implantation sites in all groups was assessed by periodic acid-Schiff (PAS) and H&E stainings.

### *In-vivo* tracking of intrauterine-perfused mesenchymal stem cells

Endometrial stromal cells were isolated from the non-pregnant CBA/J females as above and cultured in 75-cm flasks to reach 80% confluency. The cells were then labeled with 10 μm of EdU for 24 h according to the manufacturer’s instruction (Click-iT^®^ EdU Imaging Kit, Invitrogen, USA). EdU-labeled cells were harvested by using a solution of trypsin–EDTA and washed in PBS, and then approximately 5 × 10^5^ labeled cells in a total volume of 10 µL were perfused in the right horn of the uterus of three CBA/J female mice. The upper one-third of the right and left uterine horns were then collected after 2, 5, and 12 days post-perfusion for histological examination. Tissues were then processed as described elsewhere (35). In brief, tissues were fixed in a cold solution containing 2% formaldehyde and 0.002% picric acid in a 0.1 M phosphate buffer (pH 7.2) for 4 h. They were then immersed overnight in 30% sucrose prepared in 0.1 M of phosphate buffer. The specimens were embedded in Killik OCT compound embedding medium (Bio-Optica, Milan, Italy) and stored at −80°C until needed. The fixed frozen tissue specimens were sectioned at a thickness of 5 µm, mounted onto Polysine-coated slides (Hangzhou, China), and air-dried for 1 h. Following PBS rinses and tissue permeabilization with 0.5% Triton X-100, the tissues were washed and underwent EdU staining according to the manufacturer’s protocol. The sections were treated with a freshly prepared Click-iT reaction cocktail for 30 min at room temperature in the dark, followed by staining with Hoechst 33342 for nuclear staining. Slides were then mounted with 50:50 PBS–glycerol, and images were taken with an Olympus BX51 fluorescent microscope (Olympus, Tokyo, Japan).

### Quantification of regulatory T cells

On day 13.5 of pregnancy, the inguinal, renal, and paraaortic lymph nodes of pregnant CBA/J mice were extracted, and the frequency of Tregs in the mononuclear fraction of the lymph nodes was determined using a mouse regulatory T-cell kit from Thermo Fisher Scientific, USA. Due to the small size of paraaortic and renal lymph nodes, cell suspensions from these nodes were combined. To block FC receptors, cells were initially incubated with antibodies against CD16/32 for 15 min at room temperature. Subsequently, cells were stained with fluorescein isothiocyanate (FITC)-conjugated anti-mouse CD4 and phycoerythrin (PE)-conjugated anti-mouse CD25, followed by a 30-min incubation in the dark. The lymphocytes were then fixed and permeabilized using the Foxp3/Transcription Factor Staining Buffer Set and stained with allophycocyanin (APC)-conjugated anti-mouse/rat Foxp3 antibody. Lymphocytes were gated based on forward and side scatter parameters, and stained cells were analyzed using flow cytometry (PAS System; Partec) with data interpretation performed using FlowJo, version 10 software.

### One-way lymphocyte transformation assay

For the one-way lymphocyte transformation assay, pregnant CBA/J spleens were collected on day 13.5 of pregnancy, along with the spleens from male DBA/2 mice. These spleens were dispersed into a single-cell suspension through 70-µm meshes (BD Biosciences, Germany), and splenocytes were isolated using Ficoll-Hypaque density gradient centrifugation (GE Healthcare, Sweden). Splenocytes from DBA/2 male mice were inactivated with mitomycin C. Pregnant female CBA/J splenocytes were labeled with 5 µM of 5,6-carboxyfluorescein diacetate succinimidyl ester (CFSE) (Molecular Probes, USA) before co-culture with male DBA/2 splenocytes. 2.5 × 10^5^ pregnant female CBA/J splenocytes were co-cultured with an equivalent number of male DBA/2 splenocytes in a final volume of 200 μL in 96-well plates. Control wells contained CBA/J splenocytes without cells from the male partner. Cultures were maintained for 4 days, and proliferation was assessed using flow cytometry.

### Statistical analysis

Animals were allocated to different treatment conditions through random assignment. Results were expressed as means ± standard deviation (SD). Statistical significance was assessed using various methods: the Mann–Whitney test for non-parametric data and one-way analysis of variance (ANOVA) followed by the Tukey post-hoc test for comparisons involving three or more groups. A significance threshold of p <0.05 was adopted. All statistical analyses were conducted utilizing Prism 9.2 software.

## Results

### Isolation of ESCs and induction of *in-vitro* decidualization

**Figure 1:**
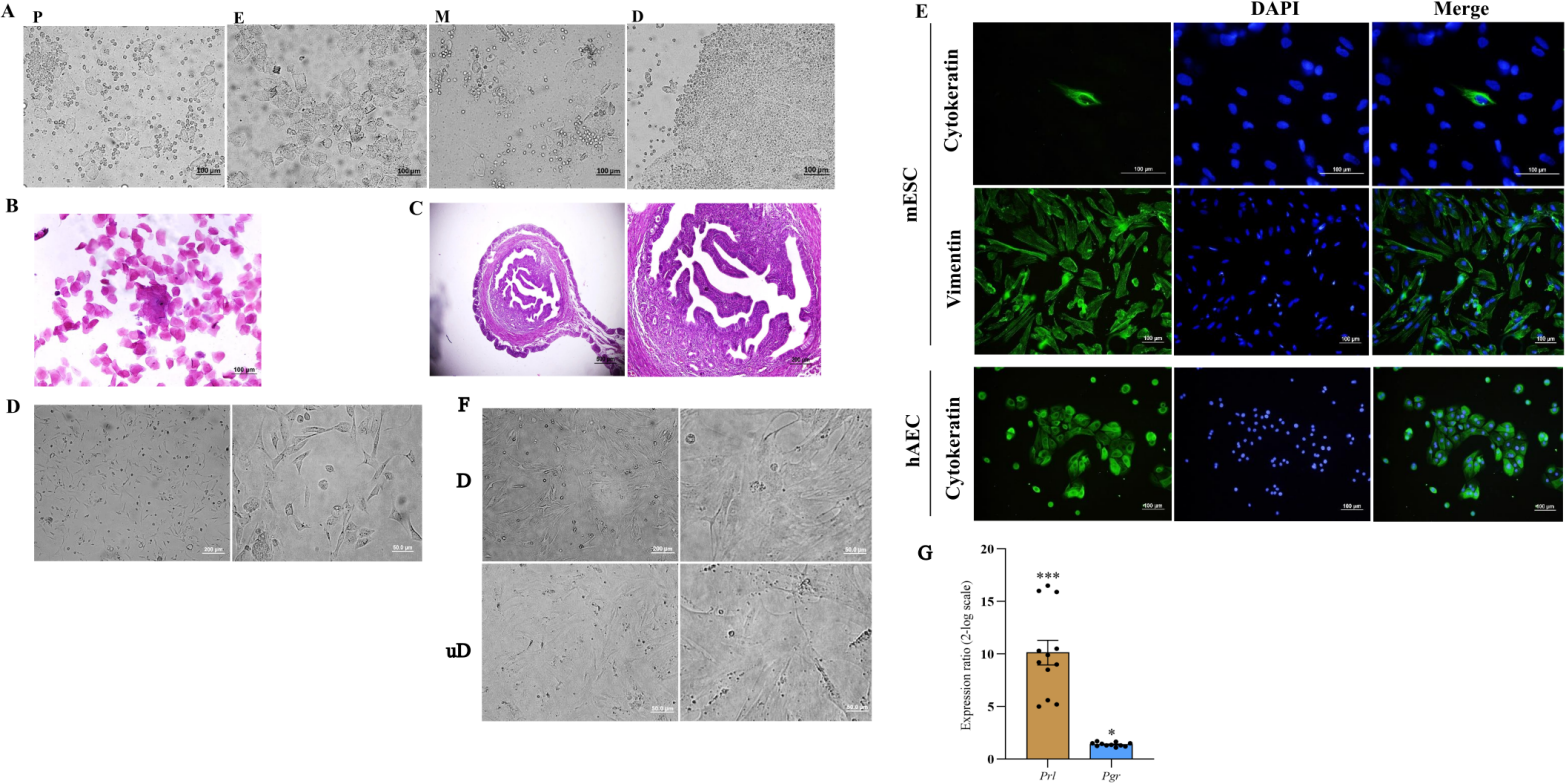
Isolation of mouse endometrial stromal cells, characterization, and *in-vitro* decidualization. The phases of the estrous cycle were determined by inspection of vaginal wet smear **(A)**, Papanicolaou staining of vaginal cells **(B)**, and histologic evaluation of uterine tissue **(C)**. Endometrial stromal cells were isolated from the uterine horn of non-pregnant CBA/J female mice and cultured **(D)**. Expression of vimentin and cytokeratin was assessed by immunofluorescence staining of isolated cells. Human amniotic epithelial cells were used as a positive control for cytokeratin staining **(E)**. *In-vitro* decidualized endometrial stromal cells transformed to a distinct morphology of rounded cells with enlarged cell size, larger nuclei, and abundant cytoplasm **(F)** and expressed Prl and Pgr mRNA **(G)**. In **(A)** P, proestrus; E, estrus; M, metestrus; D, diestrus. In **(F)** D, decidualized; unD, undecidualized. In **(E)**: mESC, mouse endometrial stromal cell; hAEC, human amniotic epithelial cell. *p < 0.05, ***p < 0.001.

The estrus phase was confirmed by a combination of vaginal wet smear examination and histologic evaluation of uterine horn sections. Vaginal cytology revealed the predominance of superficial epithelial cells appearing in some areas as epithelial sheets (**Figures 1A, B**), and histologic examination showed stromal cell proliferation, stromal congestion, and uterine gland proliferation (**Figure 1C**). ESCs were isolated from uterine horns and cultured (**Figure 1D**). Vimentin and cytokeratin immunofluorescence staining was performed to assess the purity of isolated ESCs, and our results indicated that stromal cells express vimentin but failed to express cytokeratin with purity of approximately 95%. As a positive control for cytokeratin staining, hAECs were stained with the same antibody (**Figure 1E**). Cultured ESCs were *in-vitro* decidualized using a cocktail of cAMP and MPA for 6 days. Decidualization was characterized by the differentiation of ESCs from elongated fibroblast-like endometrial stromal cells into more rounded cells with enlarged cell size, larger nuclei, and abundant cytoplasm (**Figure 1F**). Prl and Pgr mRNA levels were assessed in the in-vitro decidualized ESCs by qRT-PCR as an indication for decidualization. Compared to undecidualized ESCs, the expression of prl and pr mRNA was significantly increased in decidualized ESCs (**Figure 1G**).

### Decidualization is associated with an altered secretome profile of ESCs

**Figure 2:**
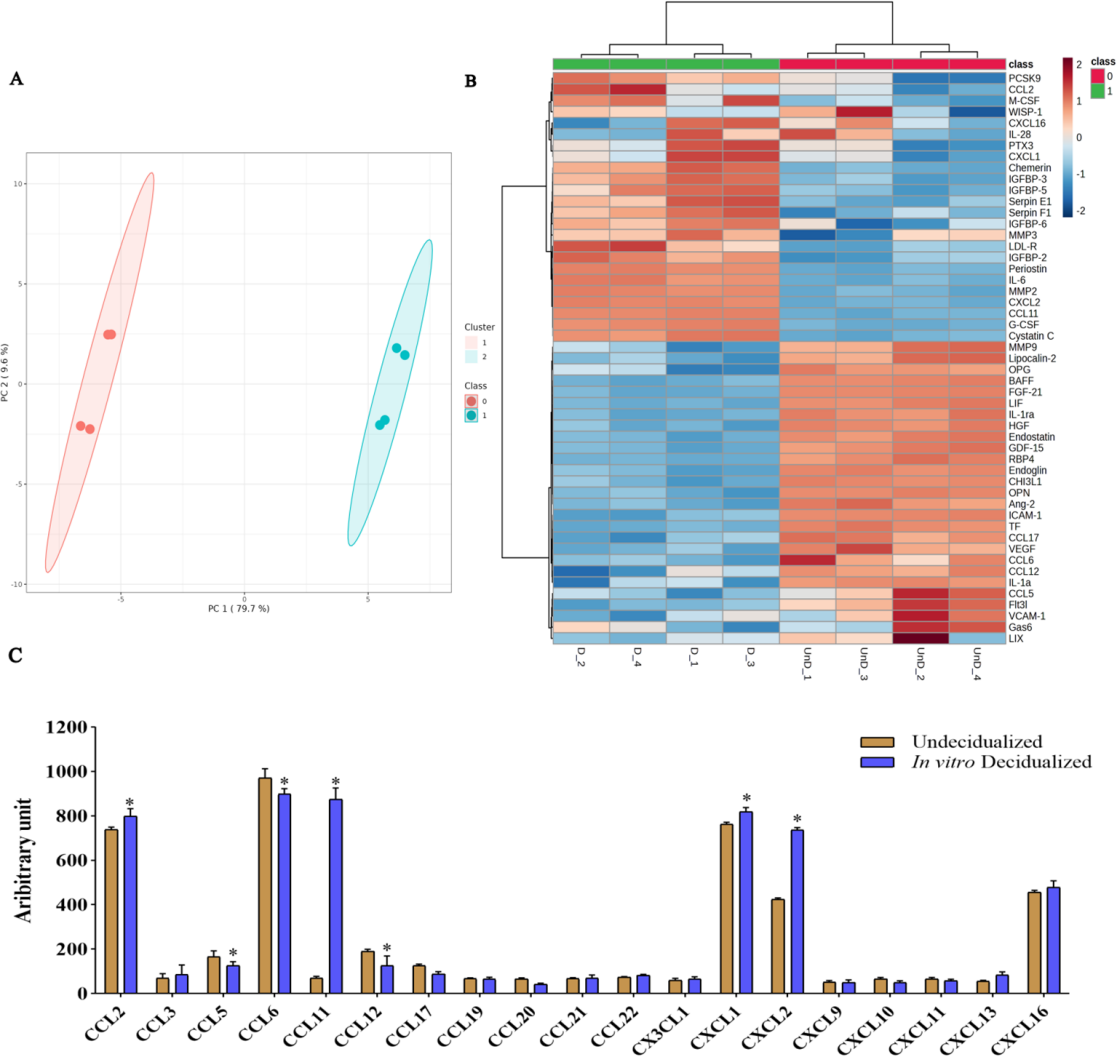
Secretome protein profiling of *in-vitro* decidualized endometrial stromal cells. Endometrial stromal cells of female CBA/J mice were *in-vitro* decidualized for 6 days, and the proteome profile of the secretome in decidualized and undecidualized cells was assessed by membrane array (n = 4). PCA showed a clear separation of proteins differentially regulated between decidualized and undecidualized endometrial cells **(A)**. A totally different pattern of protein expression in decidualized versus undecidualized ESCs was identified leading to two segregated clusters **(B)**. Production of different chemokines **(C)** was analyzed in the secretome of decidualized and undecidualized endometrial cells. *p < 0.05.

**Figure 3:**
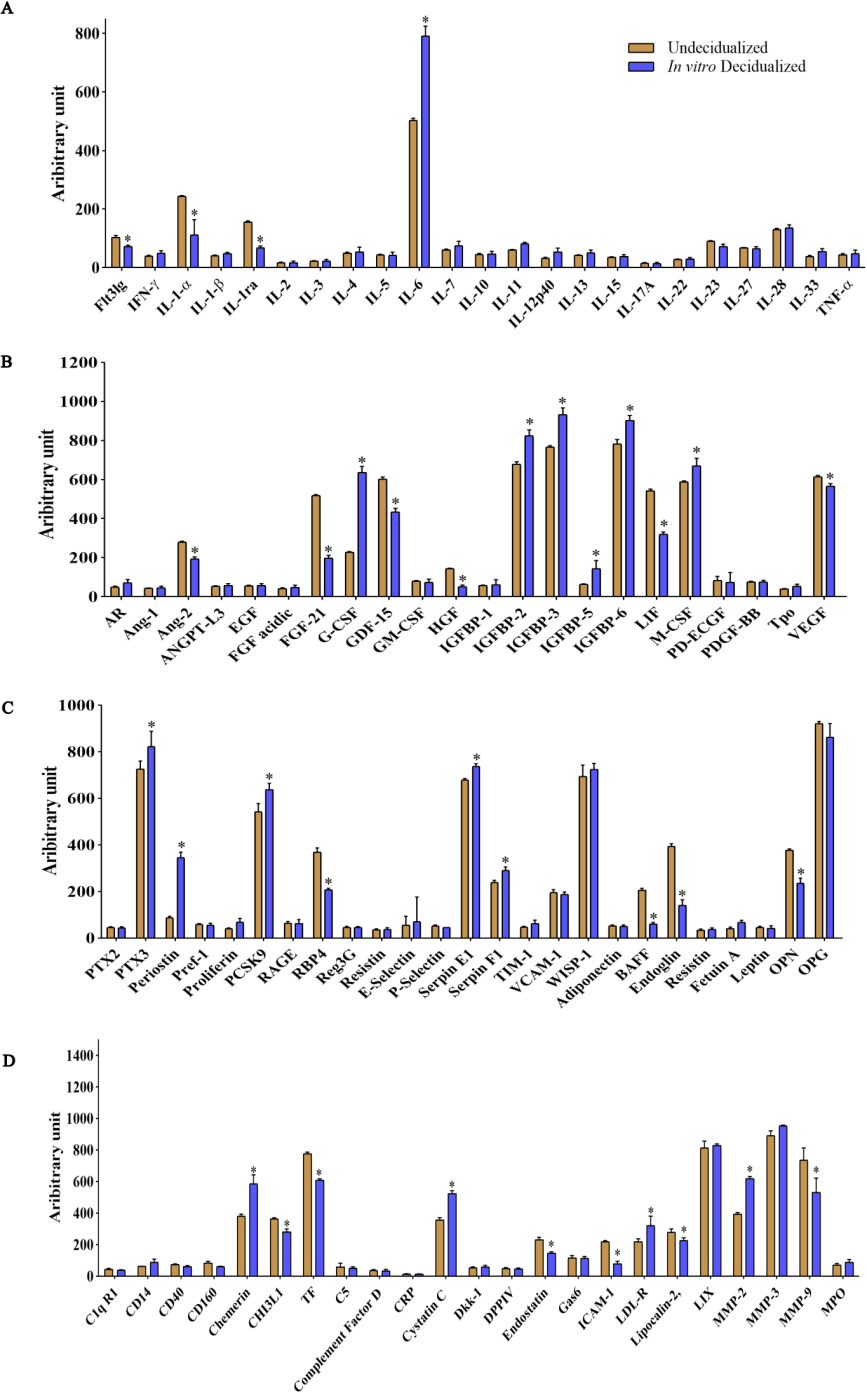
Altered expression of inflammatory cytokines, growth factors, and proteins involved in tissue remodeling and senescence in *in-vitro* decidualized endometrial stromal cells. Endometrial stromal cells of female CBA/J mice were *in-vitro* decidualized for 6 days, and the production of cytokines **(A)**, growth factors and growth factor binding proteins **(B)**, and proteins involved in different regulatory pathways including proteins involved in tissue remodeling and wound healing, hemostasis, coagulation, extracellular matrix organization, angiogenesis, inflammatory response, and senescence **(C, D)** was assessed (n = 4). *p < 0.05.

We then proceeded to proteome profiling after a 6-day period of cAMP- and MPA-induced decidualization on CBA/J endometrial stromal cells. Principal component analysis (PCA) showed a clear separation of proteins differentially regulated between decidualized and undecidualized endometrial cells (**Figure 2A**). Secretion of various chemokines and cytokines from both the decidua cells and the embryo is crucial for immune cell recruitment and activation of multiple signaling networks. So, we comparatively analyzed the secreted proteome profile of the decidualized and undecidualized ESCs using a membrane protein array system. We identified a totally different pattern of protein expression in decidualized versus undecidualized ESCs leading to two segregated clusters (**Figure 2B**). Chemokine production by ESCs was considerably altered during decidualization exemplified by significantly higher expression of chemokine (C-C motif) ligand 2 (CCL2), chemokine (C-X-C motif) ligand 1 (CXCL1), and CXCL2. Of note, secretion of CCL11 increased several folds in response to decidualization (**Figure 2C**). Decidualization was associated with a profound increase in IL-6 production (**Figure 3A**). The expression of various growth factors and their receptors in the uterus is important for the implantation process. So, we examined the secretion of various growth factors and growth factor binding proteins in decidualized endometrial cells. We found that decidualization caused an increased expression of granulocyte colony-stimulating factor (G-CSF), M-CSF, insulin-like growth factor binding protein 2 (IGFBP-2), IGFBP-3, IGFBP-5, and IGFBP-6 (**Figure 3B**). The expression of other proteins involved in different regulatory pathways including proteins involved in tissue remodeling and wound healing, hemostasis, coagulation, extracellular matrix organization, angiogenesis, and inflammatory response was also altered upon ESC decidualization. Decidualization caused a significant increase in the secretion of serpin E1, serpin F1, proprotein convertase 9 (PCSK9), periostin, pentraxin 3 (PTX3), MMP2, cystatin C, chemerin, and low-density lipoprotein receptor (LDL-R). This process is also associated with a decrease of tissue factor (TF), endostatin, intercellular adhesion molecule 1 (ICAM1), retinol-binding protein 4 (RBP4), MMP9, B-cell activating factor (BAFF), endoglin, and osteopontin (OPN) (**Figures 3C, D**).

### Decidualized ESCs display an inflammatory and senescence-associated protein signature

**Figure 4:**
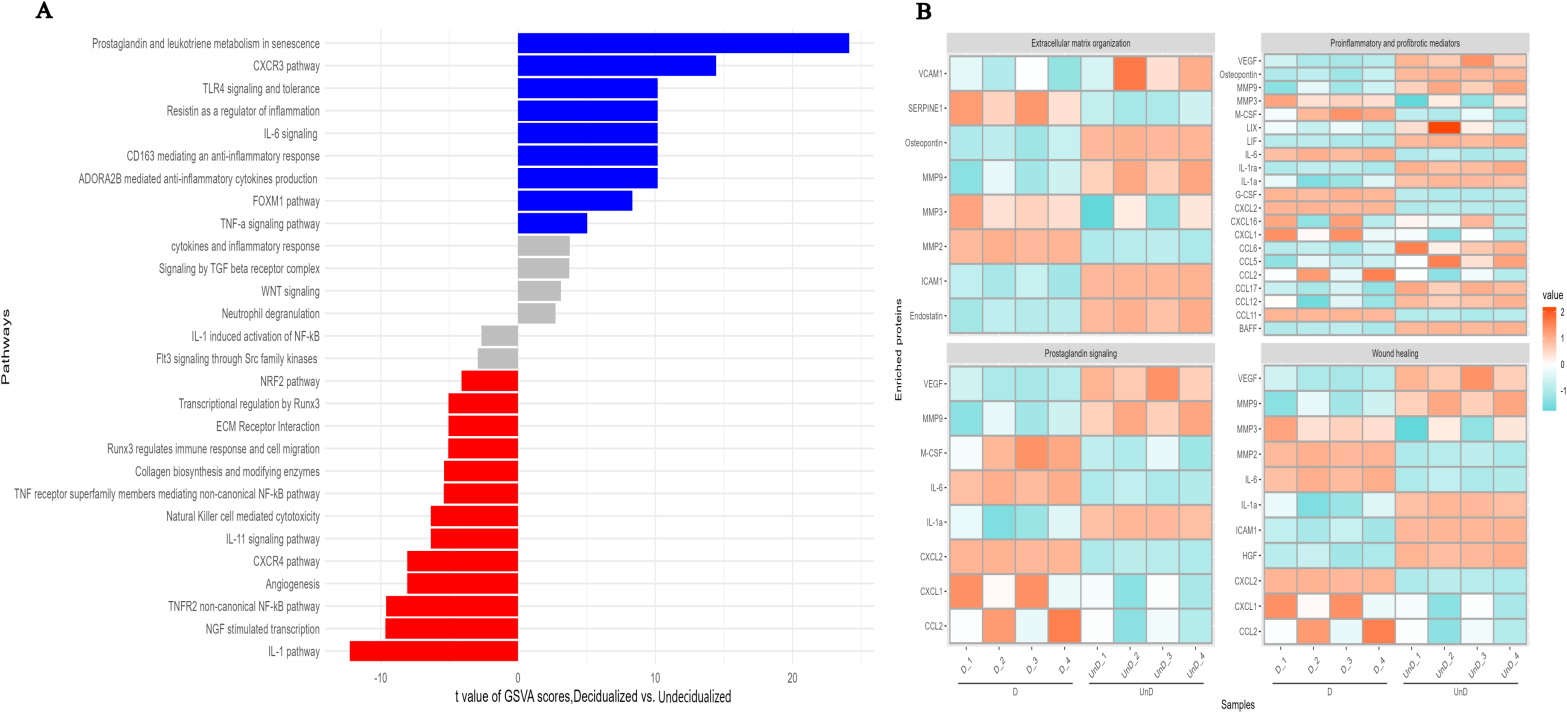
GSVA analysis of differentially expressed proteins in *in-vitro* decidualized endometrial stromal cells. Decidualization of mouse endometrial stromal cells is associated with profound changes in different signaling pathways **(A)**, extracellular organization, prostaglandin signaling, wound healing, and proinflammatory and profibrotic mediators **(B)**.

GSVA analysis of differentially expressed proteins pointed to several pathways related to decidualization. We identified higher expression of several proteins involved in inflammatory responses including IL-6, G-CSF, M-CSF, LDL-R, PTX3, SERPIN E1, CCL2, CCL11, and CXCL2 (**Figures 2C**, **3**) (36, 37) upon ESC decidualization. Notably, decidualization was associated with the downregulation of the IL-1 pathway (**Figure 4A**) suggesting a mixture of inflammatory and anti-inflammatory responses upon ESC decidualization. The cyclooxygenase 2 (COX-2)-induced prostaglandin E2 (PGE2) is actively involved in the induction of decidualization through the cAMP signaling pathway (38). Prostaglandins are active lipid molecules that are shown to have a great impact on cellular senescence (39). By focusing on senescence-associated secretory proteins including IL-6, IGFBP-2, IGFBP-3, IGFBP-4, IGFBP-6, and serpin E1, we confirmed that 6-day decidualization induces a secretome of active senescence in ESCs (**Figure 3**). Heat map analysis of wound healing, prostaglandin signaling, and extracellular matrix (ECM) remodeling proteins clearly showed differential expression of related proteins in decidualized and undecidualized ESCs (**Figure 4B**).

### Adoptive *In-utero* transfer of ESCs did not affect the number of embryos or implantation sites

**Figure 5:**
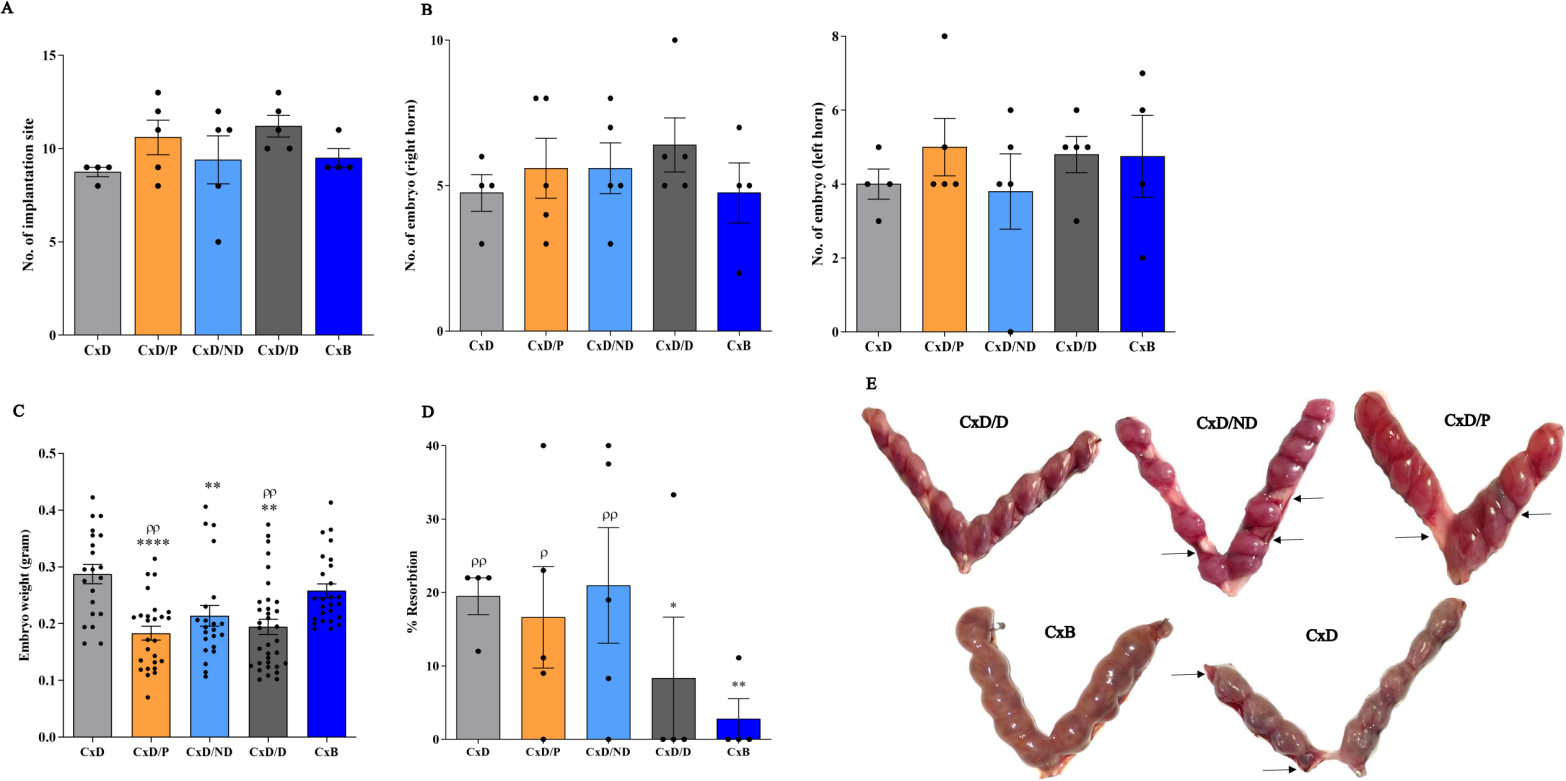
Reproduction parameters in different experimental and control groups. Five experimental groups, each consisting of four to five mice, were employed. In the C×D/D group, female CBA/J mice underwent surgical *in-utero* perfusion of decidualized ESCs, while mice in the C×D/ND and C×D/P groups received undecidualized ESCs or PBS, respectively, before mating with DBA/2 males. Two groups of female CBA/J mice were not manipulated and were mated with either male DBA/2 (C×D) or Balb/c mice (C×B). Reproduction parameters were then assessed on day 13.5 of pregnancy including the number of implantation sites **(A)**, number of embryos in the left and right horns **(B)**, embryo weight **(C)**, and resorption rate **(D, E)**. *compared to C×D, ^ρ^compared to C×B, *p < 0.05, **p < 0.01, ****p < 0.0001, ^ρ^p < 0.05, ^ρρ^p < 0.01.

Examination of the number of implantation sites showed that mice in C×D, C×D/P, C×D/ND, C×D/D, and C×B groups had an average of 10, 12.8, 10.2, 10.8, and 9.25 embryos per mouse, respectively, with no statistical difference (**Figure 5A**). To assess whether the transfer of cells to the right uterine horn could differentially affect a number of embryos in this horn, the number of embryos in the right and left uterine horns was counted separately. The results showed that the number of embryos in the right horn of the five groups (mean: 4.6, 5.6, 5.6, 6.4, and 4.75) and in the left uterine horn (mean: 5.4, 3.8, 4.75, and 4.75) did not show a statistically intergroup or intragroup significant difference (**Figure 5B**). Nonetheless, the mean fetal weight in groups C×D/P, C×D/ND, and C×D/D (0.183 g, 0.213 g, and 0.194 g) who underwent surgical procedure was found significantly lower compared to the other two control groups: C×D and C×B (mean: 0.287 g and 0.258 g, respectively) (**Figure 5C**). To give insight into the morphology of the implantation sites, PAS and H&E stainings were performed on the cross sections prepared from implantation units (decidua and placenta). PAS-reactive uterine NK cells were present in the decidua and metrial glands of all groups (**Supplementary Figure S2**).

### Adoptive *In-utero* transfer of decidualized ESCs normalized the abortion rate in CBA/J-DBA2 mating

To assess the potential effect of *in-utero* transfer of decidualized ESCs on the correction of the abortion rate, the percentage of resorption was assessed in the five groups. The mean abortion rate in the C×D, C×D/P, C×D/ND, C×D/D, and C×B groups was 20%, 16.72%, 21.44%, 8.3%, and 2.8%, respectively. The transfer of undecidualized ESCs (C×D/P) or PBS (C×D/ND) did not affect the resorption rate in DBA2-mated female CBA/J mice compared to the C×D group, while the C×D/D group showed a statistically comparable level of resorption rate with that of the C×B group (**Figures 5D, E**).

### Intrauterine perfusion of ESCs resulted in their repopulation in the uterine stroma

**Figure 6:**
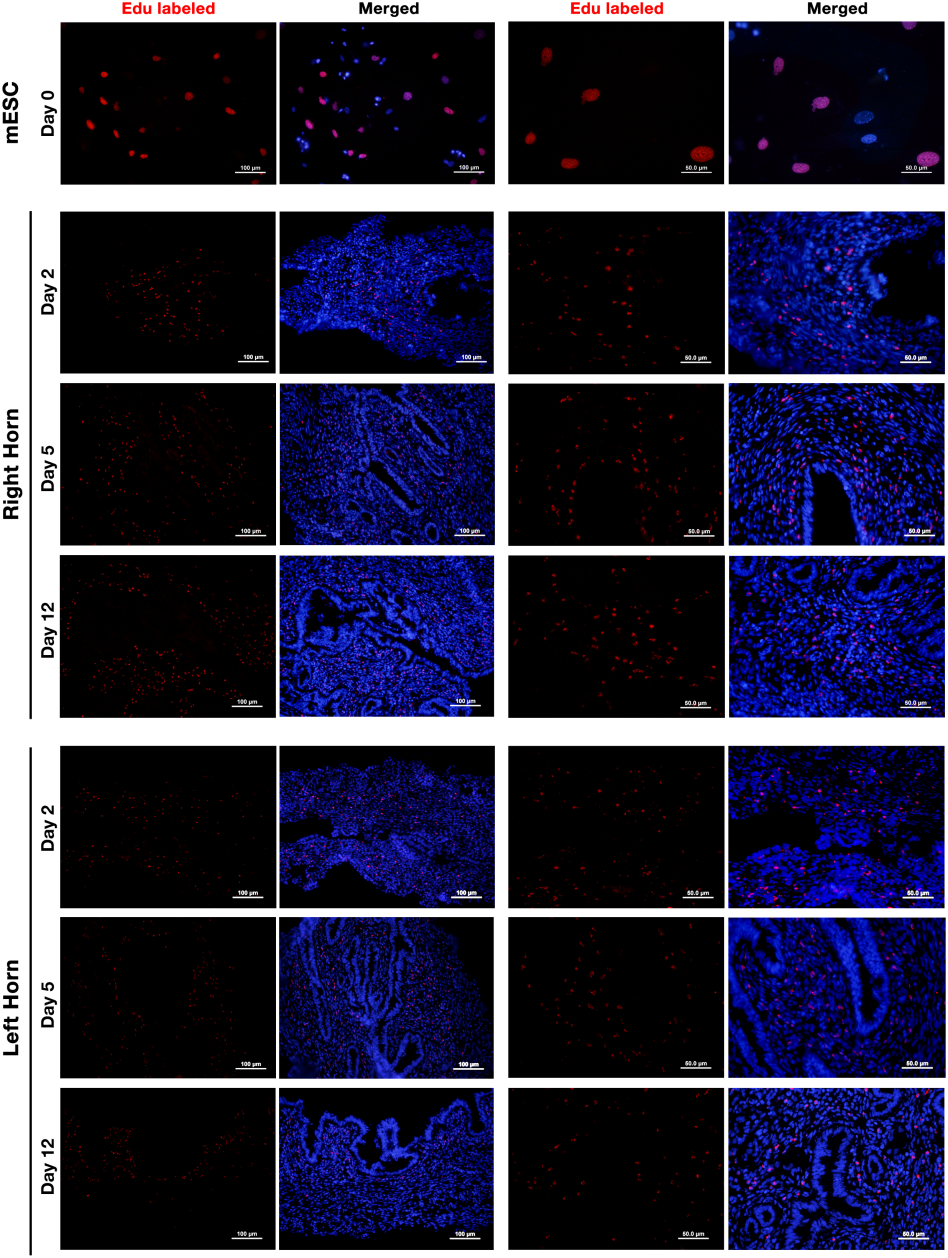
Tracking of intrauterine perfused ESCs. Endometrial stromal cells were isolated from non-pregnant CBA/J females and labeled with 10 μm of EdU for 24 h (upper panel). EdU-labeled cells were collected, and 5 × 10^5^ labeled cells were perfused in the right horn of the uterus of three CBA/J female mice. EDU-labeled ESCs were then tracked in the upper one-third of the right (middle panel) and left (lower panel) uterine horns after 2, 5, and 12 days post-perfusion by Click-iT reaction fluorescent staining followed by staining with Hoechst 33342 for nuclear staining.

EdU-labeled ESCs were perfused to the lumen of the right horn and tracked by Alexa-594 staining on days 2, 5, and 12 post-perfusion. The results showed that perfused ESCs were able to survive for at least 12 days (**Figure 6**). We did not observe a decline in the fluorescent signal of the labeled cells over the 12 days of study. Interestingly, besides the right horn of the uterus, where EDU-labeled ESCs were perfused, these cells could also be tracked in the left horn of the uterus with a lower frequency indicating dynamic traffic of the perfused cells in the uterus. Contrary to expectation, the fluorescent signal of Alexa-594 was not always confined to the cell nucleus, and cytoplasmic labeling was also observed indicating background staining. This issue had been reported earlier with the Click-iT technique without any known reason (40).

### *In-utero* transfer of decidualized but not undecidualized ESCs increased the frequency of Tregs in paraaortic and renal lymph nodes

**Figure 7:**
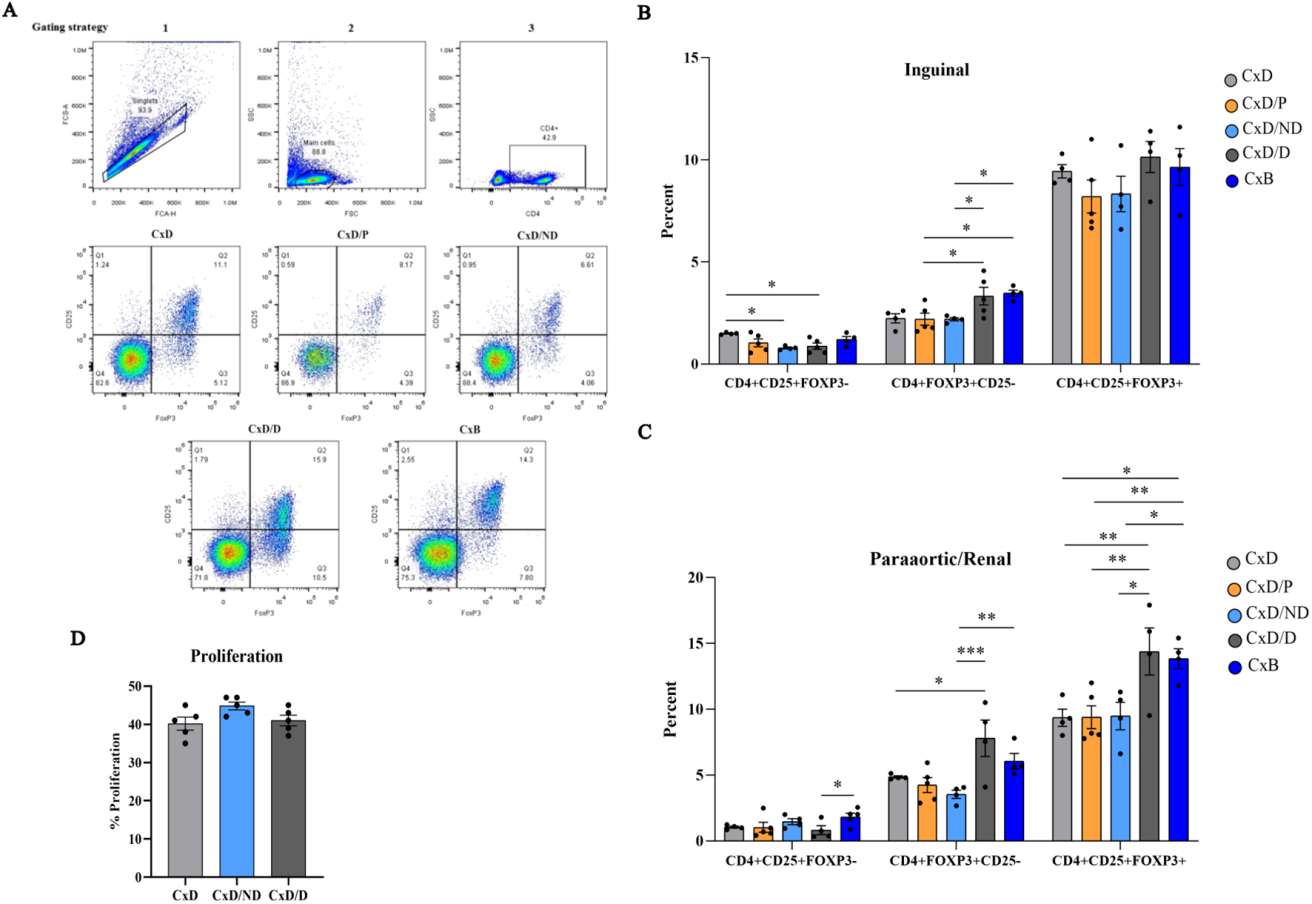
Assessment of frequency of regulatory T cells and proliferation of splenocytes. Inguinal and paraaortic/renal lymph nodes of the experimental and control groups (n = 4–5) were excised, and the frequency of CD4+CD25+FOXP3−, CD4+FOXP3+CD25−, and CD4+CD25+FOXP3+ regulatory T cells was measured by flow cytometry **(A)**. **(B, C)** The frequency of these cells in inguinal and paraaortic/renal lymph nodes, respectively. The proliferation capacity of pregnant CBA/J spleen cells in response to the stimulation with DBA/2 splenocytes in the C×D/ND and C×D/D groups was compared to the C×D group **(D)**. *p < 0.05, **p < 0.01 and ***p < 0.001

The gating strategy of Tregs is provided in **Figure 7A**. The percentage of CD4^+^CD25^+^FOXP3^−^, CD4^+^FOXP3^+^CD25^−^, and CD4^+^CD25^+^FOXP3^+^ cells was analyzed separately in five groups by flow cytometry in inguinal and paraaortic/renal lymph nodes. In inguinal lymph nodes, the percentage of CD4^+^CD25^+^FOXP3^−^ cells in C×D/ND and C×D/D mice was significantly lower than in the C×D group. The percentage of CD4^+^FOXP3^+^CD25^−^ cells in the C×D/D and C×B groups was higher than in the other groups except group C×D. No significant differences were observed between the five groups in terms of the percentage of CD4^+^CD25^+^FOXP3^+^ regulatory T cells in the inguinal lymph node (**Figure 7B**). In paraaortic/renal lymph nodes, the percentage of CD4^+^FOXP3^+^CD25^−^ cells in the C×D/D and C×B groups was significantly higher compared to the C×B/ND group, but no statistical difference was observed between the C×D/D and C×B groups. Interestingly, the percentage of CD4^+^CD25^+^FOXP3^+^ regulatory T cells in C×D/D was increased to the level observed in the C×B group and showed a statistically higher level compared to the other three groups (**Figure 7C**).

### *In-utero* transfer of ESCs did not affect the proliferative response of CBA/j splenocytes to male DBA2 splenocytes

To assess whether the immunoregulatory effects of decidualized ESCs also exerted at the systemic level, female CBA/J splenocytes on day 13.5 of pregnancy were isolated and stimulated with equal numbers of mitomycin-treated male DBA/2 splenocytes, and the proliferation rate of female CBA/J mice splenocytes was measured by CFSE flow cytometry. Data analysis did not show a significant difference in splenocyte proliferation of CBA/J mice in the C×D/ND and C×D/D groups compared to the C×D group (**Figure 7D**).

## Discussion

Despite many reports underscoring the role of immune dysregulation in inducing abortion in the CBA/J × DBA/2 model, there is strong evidence that immune dysregulation alone cannot explain abortion in this mouse model. First, mating of CBA/J females with Balb/c males, which have similar MHC haplotypes with DBA/2 mice, is considered normal. Second, reverse mating of DBA/2 females with CBA/J males is not associated with abortion (26). Third, the abortion protective effects of pre-pregnancy immunotherapy of CBA/J female mice with Balb/c splenocytes before mating with DBA/2 mice are temporary and last up to 6 weeks (41). Fourth, an organism cannot have a collection of immunological disorders but experience no adverse consequences except in pregnancy. Finally, looking at various immune dysfunctions in this mating model is like the story of Rumi’s elephant; many of the components of the immune system reported to be defective in this model are closely related to each other, and dysfunction of one component will eventually lead to the dysfunction of downstream components. Therefore, the main question that arises is what the main cause of abortion in this model is.

The endometrium plays a key role in embryo implantation and development by directing the initial phase of embryo attachment, aiding placental development and invasion, and establishing a safe microenvironment for fetal growth (30, 42). Transformation of endometrial stromal cells to the decidualized state is a fundamental step before the implantation of the human embryo. Decidualization is a multifaceted morphological transformation of endometrial stromal cells, which is associated with profound changes in the epigenetic, metabolomic, transcriptome, and proteome profiles of ESCs (4). Impaired decidualization has been associated with pregnancy loss (1, 10, 11). Decidualized human ESCs not only act as biosensors of embryo quality (43–46) but also, through extensive cross-talk, modulate the function of uterine immune cells (30, 47). Our results showed that adoptive *in-utero* transfer of decidualized, but not undecidualized, ESCs normalized the abortion rate in CBA/J-DBA2 mating comparable to the level observed in CBA/J × Balb/c mating. Interestingly, this intervention was associated with increased frequency of Tregs in the uterine draining (paraaortic and renal) but not in inguinal lymph nodes, while undecidualized ESCs failed to exert such an effect. Of note, in the C×D/D group, the frequency of unconventional Tregs (CD4^+^CD25^−^FOXP3^+^) was increased both in renal/paraaortic and inguinal lymph nodes. It has been shown that unconventional Tregs inhibited polyclonal T-cell proliferation and IFN-γ production and similarly exerted regulatory functions akin to CD25^+^ conventional Tregs (48). These results suggest that decidualization is associated with increased frequency of Tregs and that reduced frequency of these regulatory cells might not be considered as the primary cause of abortion in this model. Several lines of evidence propose that the adoptive transfer of Tregs (20, 21, 49) prevents abortion in the CBA/J × DBA/2 model. Although the robustness of these studies is beyond reasonable doubt, it can be assumed that reduced Treg frequency or impaired function is secondary to impaired decidualization. In line with this assumption, a recent study demonstrated that decidualization increased the frequency of IL-10-producing tolerogenic dendritic cells to induce Tregs (50). In humans, decidualization of ESCs inhibits the expression by these cells of chemokines that cause recruitment of TH1 and cytotoxic T cells (51). Decidualization is also associated with the huge recruitment of uterine NK cells and their transformation to regulatory phenotype with high expression of CD56 (52). More recently, Yang et al. reported a dysfunctional and spatially disordered subpopulation of decidual stromal cells with features like immune cells that cause abnormal accumulation of immune cells in the vascular hub, have aberrant increased inflammatory responses, disrupt decidual hub specification, and eventually lead to pregnancy complications in DBA/2-mated CBA/J mice (53). Collectively, these data highlight decidualized ESCs as the master regulators of the uterine immune system.

Our results showed that perfusion of EDU-labeled ESCs into the uterine lumen resulted in the appearance of EDU-positive cells in uterine stroma after 2 days, which remained there for at least 12 days. Perfusion of mesenchymal stem cells (MSCs) in the mare uterus resulted in the transient early appearance of these cells in the lumen but not in endometrial tissue (54). On the other hand, there are many reports in rodentia including mice and rats indicating that mesenchymal stem cells are easily repopulated in the endometrium for a relatively long time following intrauterine perfusion and exert functional effects (55, 56). We believe that both reports in mares and mice, although seem contradictory, are correct. The different placentation type in the mares and rodentia explains the differential localization of perfused mesenchymal stem cells, as mares have epitheliochorial placentation, while placentation in mice is of hemochorial type (57). Evidence suggests that in species with epitheliochorial placentation, the intact uterine epithelium functions as a barrier to prevent the invasion of blastocyst to the endometrial lumen. From the molecular aspect, gap junctions such as connexins 26, 32, and 43 are not detectable in the uterine epithelium of mares and pigs, whereas these molecules are readily expressed in rodents and modulated under the control of hormones (58).

We did not observe that the adoptive transfer of either decidualized or undecidualized ESCs affects the proliferative response of CBA/j splenocytes to male DBA/2 splenocytes reinforcing the fact that immunoregulation during pregnancy is mainly confined to the uterus and its draining lymph nodes.

The decidualization process in human endometrial stromal cells leads to changes in the expression of over 3,300 genes. These alterations primarily affect genes involved in regulating the cell cycle, restructuring the cytoskeleton, remodeling tissue, promoting angiogenesis, modulating the immune system, combating oxidative stress, facilitating ion and water transfer, responding to steroid hormones, and signaling chemokines, cytokines, and growth factors (53). We observed that decidualization was associated with an altered secretome profile of ESCs. Notably, we observed that decidualization caused a significant increase in IL-6 and a decrease in IL-1α production by ESCs. It is interesting to note that IL-6 knockout mice are infertile (59). IL-6 is produced cyclically by the endometrium, and its production reaches the highest level during the window of implantation (60). Of note, secretion of CCL11 increased several folds in response to decidualization. The effects of eotaxin CCL11 on extravillous trophoblast invasion and endometrial vessel remodeling have already been documented (61). Our data indicated that chemerin is upregulated during mouse ESC decidualization, which contributes to NK cell accumulation and vascular remodeling during early pregnancy (62). Analysis of ECM remodeling and senescence proteins clearly showed differential expression of related proteins in decidualized and undecidualized ESCs. During decidualization, the ECM undergoes remodeling, which involves changes in the synthesis, degradation, and organization of ECM components such as collagen, fibronectin, and proteoglycans (63). By focusing on senescence-associated secretory proteins including IL-6, IGFBP-2, IGFBP-3, IGFBP-4, IGFBP-6, and serpin E1, we confirmed that 6-day decidualization induces a secretome of active senescence in ESCs. Cellular senescence plays a crucial role in the normal process of ESC decidualization. During decidualization, a subset of cells expressing p16, a marker associated with cellular senescence, along with the activation of genes linked to senescence emerged. The severity of the initial proinflammatory response during decidualization is influenced by the quantity of pre-senescent ESCs (5, 13, 64–68).

Although our results showed that intrauterine perfusion of decidualized ESCs could significantly decrease the resorption rate in the CBA/J × DBA/2 model, correction of decidualization following cell transplantation was not directly investigated in this study. Indeed, early decidualization could be investigated in this model after mating with DBA/2 males and before when pregnancy loss occurs to define whether this is compromised in this model.

If decidualization in CBA/J mice is compromised, the immediate question that should be addressed is why mating between CBA/J females with Balb/c males is normal. Our preliminary results show that the endometrium of CBA/J mice experiences excessive inflammation upon exposure to DBA/2, but not Balb/c, mice seminal plasma, which is the main focus of our future research.

Overall, our findings showed that intrauterine perfusion of decidualized but not undecidualized ESCs in CBA/J females before mating with DBA/2 males could control abortion in this mouse model. This was associated with increased levels of regulatory T cells in the uterine drainage lymph nodes and profound alterations in proteins involved in extracellular matrix remodeling, immune cell trafficking, senescence, and inflammatory responses. These findings reinforce the pivotal role of endometrium in regulating local immune system (69) and indicate that abortion in this mouse model could not simply be attributed to the primary immune dysfunction; rather, impaired decidualization might be the cause of abortion in these mice. These findings offer a fresh outlook on reproductive immunology and fundamentally alter the approach to treating individuals experiencing recurrent miscarriages.

## Data Availability

The original contributions presented in the study are included in the article/**Supplementary Material**. Further inquiries can be directed to the corresponding authors.

## Supplementary material

Supplementary Figure 1 Perfusion of Trypan blue in to the mouse uterus. For evaluation of leakage and tracing the medium flow in the uterus, 10 µL trypan blue was perfused under a 10X operating microscope into the top of the right uterine horn at the utero-tubal junction (UTJ) **(A)** and medium flow was tracked **(B)**.

Supplementary Figure 2 H&E and PAS staining of mouse implantation unite: DBA/2-mated female CBA/J mice in different study groups were sacrificed at day 13.5 of pregnancy and implantation unites containing placenta and decidua were removed. Cross sections were prepared and stained with H&E and PAS. PAS-reactive uterine NK cells were present in the decidua (D) and metrial glands (MG) of all groups. JZ: Junctional zone, L: Labyrinth, C: Chorionic plate.
