# Supplementary figures and images for: In-utero transfer of decidualized endometrial stromal cells increases the frequency of regulatory T cells and normalizes the abortion rate in the CBA/J × DBA/2 abortion model
Source: Front Immunol. 2024 Sep 23;15:1440388. doi: 10.3389/fimmu.2024.1440388 (PMC11460546; doi:10.3389/fimmu.2024.1440388)

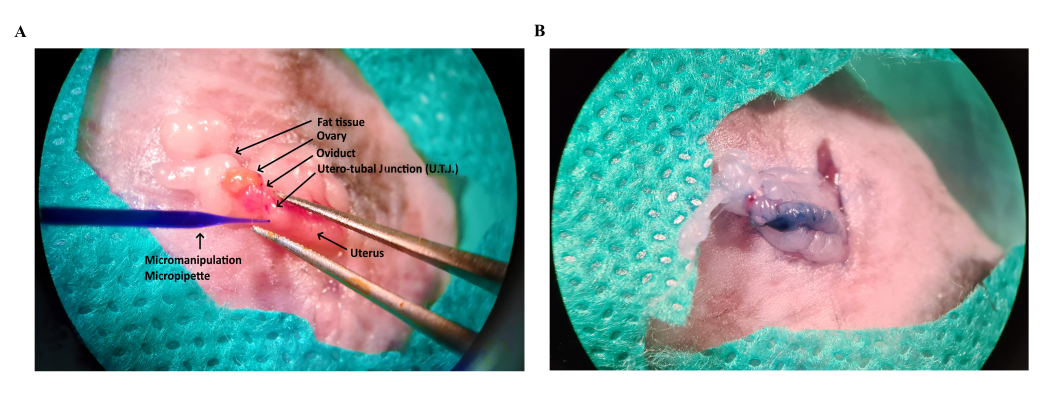

Supplement: Supplementary Figure 1 — Perfusion of Trypan blue in to the mouse uterus. For evaluation of leakage and tracing the medium flow in the uterus, 10 µL trypan blue was perfused under a 10X operating microscope into the top of the right uterine horn at the utero-tubal junction (UTJ) (A) and medium flow was tracked (B). [file Image1.tiff]

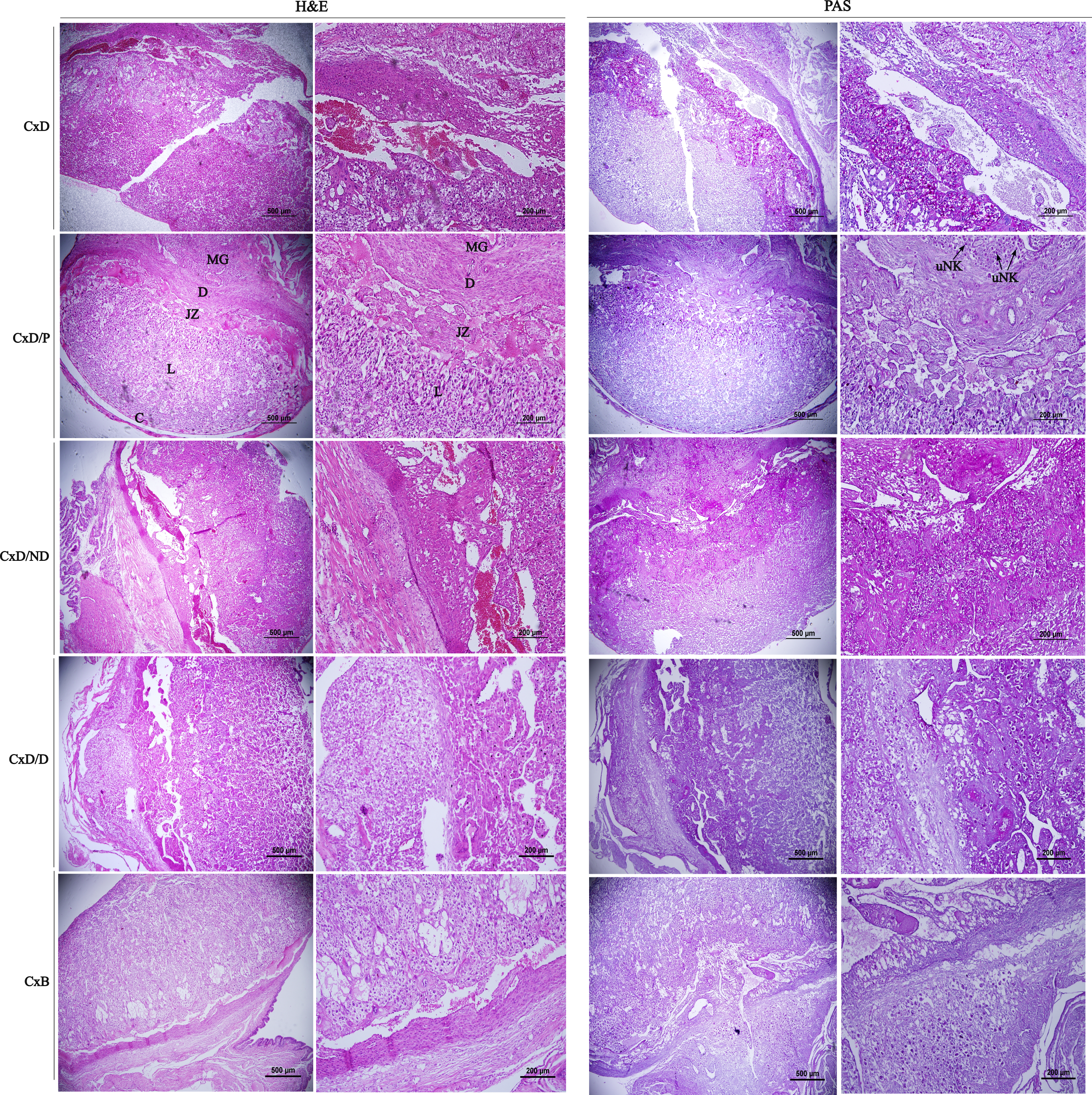

Supplement: Supplementary Figure 2 — H&E and PAS staining of mouse implantation unite: DBA/2-mated female CBA/J mice in different study groups were sacrificed at day 13.5 of pregnancy and implantation unites containing placenta and decidua were removed. Cross sections were prepared and stained with H&E and PAS. PAS-reactive uterine NK cells were present in the decidua (D) and metrial glands (MG) of all groups. JZ: Junctional zone, L: Labyrinth, C: Chorionic plate. [file Image2.tiff]
